# Acute and Chronic Effects of Cocaine on Cardiovascular Health

**DOI:** 10.3390/ijms20030584

**Published:** 2019-01-29

**Authors:** Sung Tae Kim, Taehwan Park

**Affiliations:** 1 Department of Pharmaceutical Engineering, Inje University, Gimhae 50834, Korea; stkim@inje.ac.kr; 2 Pharmacy Administration, St. Louis College of Pharmacy, St. Louis, MO 63110, USA; 3 Center for Health Outcomes Research and Education, St. Louis College of Pharmacy, St. Louis, MO 63110, USA

**Keywords:** cocaine, cardiovascular health, heart disease, acute effects, chronic effects

## Abstract

Cardiac complications resulting from cocaine use have been extensively studied because of the complicated pathophysiological mechanisms. This study aims to review the underlying cellular and molecular mechanisms of acute and chronic effects of cocaine on the cardiovascular system with a specific focus on human studies. Studies have consistently reported the acute effects of cocaine on the heart (e.g., electrocardiographic abnormalities, acute hypertension, arrhythmia, and acute myocardial infarction) through multifactorial mechanisms. However, variable results have been reported for the chronic effects of cocaine. Some studies found no association of cocaine use with coronary artery disease (CAD), while others reported its association with subclinical coronary atherosclerosis. These inconsistent findings might be due to the heterogeneity of study subjects with regard to cardiac risk. After cocaine use, populations at high risk for CAD experienced coronary atherosclerosis whereas those at low risk did not experience CAD, suggesting that the chronic effects of cocaine were more likely to be prominent among individuals with higher CAD risk. Studies also suggested that risky behaviors and cardiovascular risks may affect the association between cocaine use and mortality. Our study findings highlight the need for education regarding the deleterious effects of cocaine, and access to interventions for cocaine abusers.

## 1. Introduction

Cocaine is a tropane alkaloid compound that can be extracted from the leaves of an Andean shrub, *Erythroxylon coca*, in South America. Cocaine was originally used for local surgeries as an anesthetic agent in the 1880s, but it became a recreational drug in the 1970s. In the 1980s, there was an epidemic of cocaine use, with the number of cocaine users in the US estimated at 5.8 million in 1985 [1]. In 2016, the total number of cocaine users was estimated to be 18.2 million worldwide [2]. Approximately 34% of these cocaine users resided in North America, and 20% resided in Western and Central Europe. In the US, there were 1.5 million cocaine users aged 12 or older, representing 0.6% of the population [3]. Young adults aged 18 to 25 were the most common cocaine users (1.4%).

Cocaine may be administered by smoking, intravenous injection, nasal inhalation, or oral application. Pharmacokinetics vary by route of administration, with time to peak blood concentration ranging from 1–5 min (smoking or intravenous injection) to 60–90 min (oral administration) [4,5]. The duration of pharmacological action ranges between 5–60 min following smoking or intravenous administration, and up to 180 min following oral administration. In addition to nasal mucous membranes, cocaine absorption through other mucous membranes such as intravaginal or intrarectal mucus membranes is also possible [6]. Cocaine administration through mucous membranes results in slower onset of action, later peak concentration, and longer duration of action compared with that of smoking or intravenous administration, but faster onset of action, earlier peak concentration, and shorter duration of action than that of oral administration. Cocaine is converted into two major metabolites by plasma and liver cholinesterases: benzoylecgonine and ecgonine methyl ester. These water-soluble metabolites are excreted in the urine and are detectable in the urine for 24 to 36 h after intake.

Cocaine is categorized as a Schedule II substance under the Controlled Substances Act. Drugs or substances in this schedule have a high potential for abuse, which may lead to severe psychological or physical dependence. Cocaine abuse can result in a range of adverse health outcomes. About 0.9 million U.S. adults had a cocaine use disorder in 2014 [7]. Approximately 40% of all emergency department visits related to drug misuse and abuse were attributed to cocaine [8].

Prior studies have consistently reported the deleterious effects of cocaine use/abuse on the cardiovascular system. Cocaine-related cardiac complications include acute conditions such as arrhythmia and acute myocardial infarction (MI), as well as chronic conditions such as cardiomyopathy and coronary artery disease (CAD). Cocaine-induced cardiotoxicity can result in sudden death. In addition, previous studies have explored the complicated pathophysiological mechanisms of cocaine cardiotoxicity. Herein, we first review the cellular and molecular mechanisms of cocaine in the cardiovascular system to obtain a better understanding of its acute and chronic effects on the heart and blood vessels. Furthermore, we discuss recent evidence from human studies that examined cocaine-associated changes in the cardiovascular system. As such, our review includes recent clinical studies that have been published in the past 10 years (from September 2008 through September 2018) retrieved from the Medline database, and several other important clinical studies published before September 2008.

## 2. Pathophysiological Mechanisms of Cocaine on Cardiovascular Health

**Figure 1:**
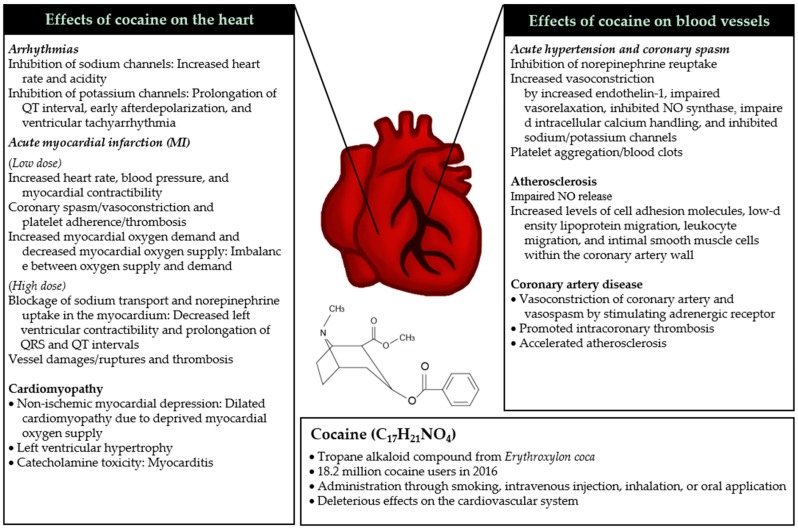
Effects of cocaine on cardiovascular health. Use of cocaine (bottom) results in both acute (italic) and chronic (normal) changes in the heart (left) and blood vessels (right). (Note: Cocaine often induces cardiac condition(s) (e.g., acute myocardial infarction (MI) and coronary artery disease) by affecting the heart and vessels simultaneously).

Cocaine stimulates the sympathetic nervous system by inhibiting reuptake of norepinephrine, dopamine, and serotonin by interacting with each transporter, leading to exaggerated, prolonged sympathetic nervous system activity [9,10]. Cocaine also blocks sodium/potassium channels, which induces abnormal, depressed cardiovascular profiles [11]. In particular, concurrent cocaine and alcohol abuse significantly increases cocaine levels in the blood, leading to increased, prolonged cardiovascular risks [12]. Previous studies have reported that use/abuse of cocaine is associated with increased risk of subsequent cardiovascular complications such as hypertension, coronary spasm, arrhythmias, MI, cardiomyopathy, atherosclerosis, and CAD [13], as summarized in Figure 1. In this section, we summarize the acute and chronic pathophysiological mechanisms of cocaine on cardiovascular health.

### 2.1. Mechanisms of Acute Toxicity

#### 2.1.1. Acute Hypertension and Coronary Spasm

Acute coronary events usually occur within minutes to hours after cocaine administration. Cocaine stimulates the adrenergic system by binding to norepinephrine transporters, resulting in increased norepinephrine effects at postsynaptic receptor sites. Blocking norepinephrine reuptake induces tachycardia and hypertension, which increases myocardial oxygen demand and reduces myocardial oxygen supply by vasoconstriction [11,14,15]. As such, cocaine induces sympathetic effects on the cardiovascular system by enhanced inotropic and chronotropic effects through increased vasoconstriction. In particular, cocaine induces acute hypertension due to increased vasoconstriction induced by increased endothelin-1 [16], impaired acetylcholine-induced vasorelaxation [17], inhibition of nitric oxide synthase [18], impaired intracellular calcium handling [19], and inhibition of sodium/potassium channels [20] as determined by cellular and molecular analytical approaches [11]. In addition, acute vessel damage induces platelet aggregation/blood clots through increased fibrinogen and von Willebrand factor, leading to acute heart damage due to reduced blood flow [21]. Taken together, cocaine induces acute hypertension, coronary spasm, which may lead to subsequent myocardial infarction.

#### 2.1.2. Arrhythmias

Previous studies have shown that cocaine inhibits cardiac ion channels such as sodium channels and potassium channels [22]. The upstroke of action potential was shown to be delayed in response to sodium channel blockade, which is modulated by heart rate and acidity. Increased heart rate and acidity boost the effect of cocaine on sodium channels [23,24]. Inhibition of sodium channels is intensified when cocaine is abused or when cocaethylene is formed after administration of cocaine with alcohol [25,26]. Cocaine effects on potassium channel blockade result in prolonged QT interval, early afterdepolarization, and ventricular tachyarrhythmia [27,28]. Similar to effects on sodium channels, cocaine abuse or cocaine use with alcohol exacerbates inhibition of potassium channels and QT prolongation [29]. In addition, cocaine administration increases body temperature, resulting in hyperthermia. Cocaine overdose can induce cardiac arrhythmias and result in an impaired electrocardiographic profile, which may be related to the increased prevalence of cocaine-associated mortality in hot weather and in crowded circumstances [30,31]. In addition to these factors, cardiac arrhythmias may be affected by other factors such as catecholamine excess and calcium channel blockade. Acidosis and electrolyte abnormalities can also modulate cardiac arrhythmias. [23]. As such, cocaine-induced cardiac arrhythmias can be generated via many mechanisms in cocaine users.

#### 2.1.3. Acute Myocardial Infarction

Mechanisms of acute MI resulting from cocaine use are multifactorial. Cocaine and its metabolites are sympathomimetic agents [32] and induce local anesthetic effects [11]. At low doses, cocaine-induced sympathetic effects increase heart rate, blood pressure, and myocardial contractibility, leading to increased myocardial oxygen demand [33]. Cocaine also enhances coronary spasm/vasoconstriction and platelet adherence/thrombosis, leading to reduced myocardial oxygen supply [34]. Thus, an imbalance between oxygen supply and demand results in MI [35]. At high doses, cocaine-induced local anesthesia results in decreased left ventricular (LV) contractibility and prolongation of QRS and QT intervals in electrocardiograms by blocking sodium transport and norepinephrine uptake in the myocardium [4]. In vessels, cocaine contributes to MI by increasing endothelin-1 [36] and reducing nitric oxide production in endothelial cells [37]. When vessels are stressed, acute damages/ruptures can occur, which promotes thrombosis by increasing platelet activity/aggregation [38,39] and elevating fibrinogen levels [40] and plasminogen activator inhibitor activity [41,42]. These cellular and molecular cascades result in reduced cardiac blood flow, leading to acute MI and possibly atherosclerosis and coronary thrombosis in the long term [43,44]. As such, cocaine induces acute MI by directly affecting myocardial tissues in the heart and indirectly enhancing thrombosis in vessels.

### 2.2. Mechanisms of Chronic Toxicity

#### 2.2.1. Cardiomyopathy

Cocaine causes systolic dysfunction or LV failure, which results from reduced ejection fraction and an enlarged left ventricular chamber [45]. Cocaine administration reduces myocardial contractility and ejection fraction [46] and enhances left ventricular end-diastolic pressure and end-systolic volume [47,48,49]. It may cause non-ischemic myocardial depression, leading to dilated cardiomyopathies such as Takotsubo cardiomyopathy, a type of non-ischemic cardiomyopathy [50]. Previous studies reported that cocaine-induced cardiomyopathy, especially dilated cardiomyopathy [51,52], resulting from deprivation of myocardial oxygen supply despite increased demand for oxygen, leads to reduced coronary blood flow. Dilated cardiomyopathy is the most common consequence of long-term cocaine use and can lead to several complications including heart failure and heart-valve defects [53]. Chronic abuse of cocaine is associated with left ventricular hypertrophy [54]. In addition, catecholamine toxicity from chronic cocaine use was shown to be associated with myocarditis [55], which was related to increased local immune reactions and myocardial necrosis [56].

#### 2.2.2. Atherosclerosis

Coronary atherosclerosis often occurs in young cocaine users [24,57] or cocaine users with other cardiovascular diseases (e.g., MI) [58]. According to previous studies, cocaine impairs nitric oxide release from endothelial cells [59,60]. In addition, cocaine increases levels of cell adhesion molecules (e.g., intracellular adhesion molecule-1 (ICAM-1), cluster of differentiation 54 (CD54), vascular cell adhesion molecule-1 (VCAM-1), endothelial leukocyte adhesion molecule-1 (ELAM-1)), low-density lipoprotein migration, and leukocyte migration in blood vessels [61]. Moreover, intimal smooth muscle cells within the coronary artery wall increase [24,62], presumably leading to progression of atherosclerosis and potential sudden cardiac death [63]. Based on immunological studies, mast cells in plaques may contribute to atherosclerosis, vasospasm, thrombosis, and sudden death [57,59,64]. Briefly, proteolytic substances released from mast cells accelerate atherosclerosis by degrading and facilitating uptake of low-density lipoprotein cholesterol by macrophages [65,66]. Histamine released from mast cells increases endothelial permeability, which leads to leukocyte migration [67]. As such, cocaine has complex effects on endothelial cell dysfunction, facilitates low-density lipoprotein and leukocyte migration, and increases intimal smooth muscle cells, all of which contribute to atherosclerosis in long-term users.

#### 2.2.3. Coronary Artery Diseases

Chronic cocaine use causes repetitive damages to the heart and vessels by interacting with norepinephrine transporters [68]. Alpha-2 adrenergic receptors induce vasoconstriction of coronary arteries through contraction of vascular smooth muscle cells [34], leading to prothrombotic effects caused by increased von Willebrand factor [21]. Cocaine induces vasospasm through stimulation of adrenergic receptors on coronary arteries [69]. Cocaine also promotes intracoronary thrombosis [70,71] through increased von Willebrand factor release, increased levels of endothelial tissue factor, an important factor in pathogenesis of acute coronary syndrome (ACS), decreased levels of tissue factor pathway inhibitor [72], and accelerated atherosclerosis due to endothelial cell dysfunction [60]. In addition, long-term use of cocaine induces endothelial injury, vascular fibrosis [73,74], and subsequent vessel wall weakening [75], resulting in apoptosis of vascular smooth muscle cells and cystic medial necrosis [76,77]. According to previous reports, cocaine sometimes induces coronary and carotid aortic dissections [78,79,80]. Thus, cocaine causes coronary artery diseases through multifactorial mechanisms including vasoconstriction, intracoronary thrombosis, and accelerated atherosclerosis.

## 3. Cocaine Cardiotoxicity in Human Studies

Cocaine-induced cardiotoxicity can result in deleterious effects on the heart and vessels through multifactorial pathophysiological mechanisms, as described above. In this section, we focus on recent human studies published in the past 10 years, retrieved from the Medline database. Table 1 presents these studies that examined the association of cocaine use with both acute and chronic cardiovascular diseases and mortality.

### 3.1. Acute Effects of Cocaine

A number of studies have reported a possible link between cocaine use and acute cardiovascular conditions such as acute hypertension, arrhythmia, coronary artery aneurysms (CAAs), and acute MI. Because the study populations and data sources varied across the studies, the findings of these studies should be interpreted carefully in the context of each individual study.

Kozor et al. [81] in Australia compared blood pressure, aortic stiffness, and LV mass in cocaine users with those in cocaine non-users. The authors recruited 20 regular cocaine users aged 37 ± 7 years (85% male) and 20 control subjects aged 33 ± 7 years (95% male). This study defined regular cocaine use as using cocaine at least monthly during the year prior to when the study was conducted. The study findings showed that cocaine users had higher systolic blood pressure (134 ± 11 vs. 126 ± 11 mm Hg), increased aortic stiffness, and greater LV mass (124 ± 25 vs. 105 ± 16 g) compared with cocaine non-users.

In addition, Sharma et al. [43] retrospectively reviewed electrocardiogram (ECG) recordings of cocaine-dependent subjects to examine cardiotoxicity of cocaine use. The ECGs were collected from 97 cocaine-dependent subjects aged 50 ± 4 years (86% male) in a comprehensive academic health center and 8513 cocaine-non-using subjects aged 52 ± 5 years (46% male) participating in the Atherosclerosis Risk in Communities (ARIC) study. The authors found significant effects of cocaine use on early repolarization (odds ratio (OR) = 4.92, 95% confidence interval (CI): 2.73–8.87), bradycardia (OR = 3.02, 95% CI: 1.95–4.66), severe bradycardia (OR = 5.11, 95% CI: 2.95–8.84), and heart rate (B weight = −5.84, 95% CI: −7.85 to −3.82). Recently, there was a case report of Mobitz type II atrioventricular (AV) block associated with cocaine use [82]. This case occurred in a 55-year-old female who presented with chest pain after cocaine use.

Satran et al. [83] investigated the prevalence of CAAs among cocaine users undergoing coronary angiography using a database from a medical center in the US. The study population included 112 patients with a history of cocaine use aged 44 ± 8 years (79% male) and 79 patients with no history of cocaine use aged 46 ± 5 years (61% male). Based on the finding that cocaine users had a significantly higher CAA compared with cocaine non-users (30.4% vs. 7.6%, respectively), the authors concluded that cocaine users were likely to be at increased risk of acute MI.

Several studies examined the association between cocaine use and MI. Gupta et al. [84] examined the incidence of acute ST elevation myocardial infarction (STEMI), cardiogenic shock, multivessel CAD, and in-hospital mortality in the cocaine group (*n* = 924) compared with the non-cocaine group (*n* = 102,028) among patients admitted within 24 h of acute MI. This study used data from the National Cardiovascular Data Registry Acute Coronary Treatment and Intervention Outcomes Network Registry-Get With The Guidelines (ACTION Registry-GWTG) program. Compared with the non-cocaine group, the cocaine group was younger (average age: 50 (44–56) vs. 64 (54–76)), had a higher proportion of men (80% vs. 65%) and African-Americans (45% vs. 9%), and fewer traditional cardiovascular risk factors such as hypertension (65% vs. 71%), dyslipidemia (42% vs. 59%), previous coronary bypass (6% vs. 14%), and previous revascularization (24% vs. 31%). Gupta et al. [84] found higher percentages of STEMI (46% vs. 40%) and cardiogenic shock (13% vs. 4%) in cocaine users although their percentage of multivessel CAD was lower (53% vs. 65%) compared with cocaine non-users. In-hospital mortality was similar between the two groups (OR = 1.00, 95% CI: 0.69–1.44). Another study conducted by Salihu et al. [85] included pregnant women aged 13–49 years to examine the association of cocaine use with incidence of acute MI or cardiac arrest during pregnancy or childbirth. This retrospective study used data from January 2002 through December 2014 from the National Inpatient Sample (NIS), a large public inpatient database in the U.S. The study results showed that cocaine users (*n* = 153,608) were at higher risk for acute MI or cardiac arrest compared with drug non-users (*n* = 56,882,258), with adjusted OR of 1.83 (95% CI: 1.28–2.62). Some studies showed that the association between cocaine use and MI was affected by some confounders such as cardiac risk factors and risky behaviors. For example, Aslibekyan et al. [86] conducted a retrospective study examining the prevalence of MI among civilian non-institutionalized US adults. Using data from the National Health and Nutrition Examination Survey (NHANES), their study included two different age groups for their study population: (a) individuals aged 18–59 years (*n* = 11,993, 46% male) and (b) those aged 18–45 years (*n* = 9337, 39% male). Although Aslibekyan et al. [86] found no association between cocaine use and MI in the 18–59 age group, cocaine use of >10 lifetime instances was significantly associated with MI in the 18–45 age group after adjusting for age (aged-adjusted OR = 4.60, 95% CI: 1.12–18.88). This association was affected by cardiac risk factors (e.g., smoking status, history of diabetes, hyperlipidemia, and hypertension) in the multivariate-adjusted model (OR = 3.84, 95% CI: 0.98–15.07). Another retrospective study by Gunja et al. [87] examined the association of cocaine use with MI and 1-year all-cause mortality. The study included veterans with CAD who underwent coronary catheterization between October 2007 and September 2014 using the Veterans Affairs database. Compared with the non-cocaine group (*n* = 118,953), the cocaine group (*n* = 3082) was younger (median age: 58 vs. 65), more likely to be African-American (59% vs. 11%) and had fewer traditional cardiac risk factors. After adjusting for cardiac risk factors, cocaine use was significantly associated with MI (hazard ratio (HR) = 1.40, 95% CI: 1.07–1.83); however, this association became attenuated after controlling for risky behaviors in the sequential multivariable model (HR = 1.17, 95% CI: 0.87–1.56).

In summary, prior studies have reported that cocaine use was associated with acute cardiovascular conditions such as elevated blood pressure, (severe) bradycardia, CAAs, and acute MI. These findings are consistent with earlier studies documenting cocaine-related MI [59,98]. Of note, the studies in this review suggest that the association between cocaine use and MI might be confounded by cardiac risk factors or risky behaviors. Accordingly, the risk of MI among cocaine users needs to be understood in the context of risk factors and risky behaviors.

### 3.2. Chronic Effects of Cocaine

Several studies examined whether cocaine use was associated with chronic cardiovascular conditions such as cardiomyopathy (e.g., LV hypertrophy), subclinical atherosclerosis, and CAD. In this section, we present the characteristics of each study along with the study findings. We interpreted the results with consideration of study populations and data sources.

Maceira et al. [45] found that cocaine abusers had increased LV end-systolic volume, LV mass index, and right ventricular (RV) end-systolic volume, with decreased LV ejection fraction and RV ejection fraction. The study participants were 94 cocaine abusers aged 37 ± 7 years (86% male) attending a rehabilitation clinic for the first time. They were compared with an age- and gender-matched healthy group. As previously mentioned, Kozor et al. [81] also showed greater LV mass among regular cocaine users compared with cocaine nonusers.

Furthermore, several previous studies examined the association between cocaine use and CAD. The effects of cocaine on subclinical CAD were examined using different CAD surrogate markers [84,88,89,90,91]. For example, Arora et al. [88] examined the presence of subclinical CAD using carotid intima media thickness (CIMT) as a surrogate marker. This cross-sectional study included 33 Caucasian adults aged 37 ± 9 years who used cocaine (33% male). Their findings suggested no association between chronic cocaine use and subclinical CAD measured by CIMT. Another study conducted by Bamberg et al. examined the association of cocaine use with CAD and ACS using coronary computed tomography (CT) [89]. The study subjects were patients who presented to the emergency department (ED) with acute chest pain. In this nested matched cohort study, there were 44 patients in the cocaine group aged 46 ± 7 years (86% male) and 132 patients in the age- and gender-matched non-cocaine group. The authors found no significant association between cocaine use and coronary stenosis, but found a significant association between cocaine use and ACS (OR = 5.79, 95% CI: 1.24–27.02). Chang et al. [90] conducted another cross-sectional study that included patients who received coronary computerized tomographic angiography (CTA) for evaluation of CAD in the ED. The patients were at low- to intermediate-risk for ACS. Of these patients, cocaine users were aged 46±6 years (*n* = 157, 58% male) while the non-cocaine group was aged 48 ± 9 years (*n* = 755, 40% male). Chang et al. [90] found no association between repetitive cocaine use and coronary calcifications or between recent cocaine use and CAD. As noted previously, Gupta et al. [84] investigated the incidence of multivessel CAD in addition to STEMI and cardiogenic shock between the cocaine group (*n* = 924) and the non-cocaine group (*n* = 102,028) among patients admitted within 24 h of acute MI. They found a lower percentage of multivessel CAD among cocaine users than cocaine nonusers (53% vs. 65%), although the percentages of STEMI (46% vs. 40%) and cardiogenic shock (13% vs. 4%) were higher. In contrast to these studies, a study by Lai et al. [91] found a higher risk for subclinical CAD among cocaine users compared with cocaine non-users (propensity score-adjusted prevalence ratio (PR) = 1.27, 95% CI: 1.08–1.49). The subjects in the study by Lai et al. [91] were African Americans aged 45 years (Interquartile range (IQR): 40–50), of whom 60% were males. Approximately 67% of the subjects were HIV-positive. In this cross-sectional study, subclinical CAD was defined by the presence of coronary artery calcium (CAC) detected by non-contrast CT and/or coronary plaque detected by contrast-enhanced CT angiography (CCTA). Chronic cocaine users were at significantly higher risk for the presence of CAC (propensity score-adjusted PR = 1.26, 95% CI: 1.05–1.52), any coronary stenosis (propensity score-adjusted PR = 1.30, 95% CI: 1.08–1.57), and calcified plaques (propensity score-adjusted PR = 1.37, 95% CI: 1.10–1.71), in addition to subclinical CAD. Another study conducted by Lucas et al. [92] showed a significant association between cocaine use and carotid plaque formation. More than 90% of subjects in this study were African Americans. Cocaine non-users were aged 46 years (IQR: 41–53), and 67% were male. Past cocaine users were aged 51 years (IQR: 46–54), and 66% were male. Current cocaine users were aged 49 years (IQR: 45–52), and 75% were male. Of the study subjects, approximately 66% were HIV-positive. Compared with cocaine non-users, both past cocaine users and current users had approximately three-fold higher odds of having carotid plaques at baseline (OR = 3.3, 95% CI: 1.5–7.3 and OR = 2.7, 95% CI: 1.3–5.5, respectively).

In summary, cocaine was reported to be associated with high risk for cardiomyopathy characterized by LV hypertrophy [45,81] and ACS [89]. In particular, one study found an approximately six-fold higher risk for ACS among cocaine users [89]. However, studies have reported inconsistent findings regarding association between cocaine use and subclinical CAD. Some studies found no association of cocaine use with coronary calcifications [88,89,90]. This result is consistent with findings in the Coronary Artery Risk Development in Young Adults (CARDIA) study which examined the association between cocaine exposure and prevalence of coronary calcification by including over 3000 participants [99]. The CARDIA study reported no relationship between cocaine exposure and coronary calcium after adjusting for age, sex, ethnicity, socioeconomic status, family history, tobacco use, and alcohol use. However, Lai et al. [91] reported that cocaine use was associated with subclinical coronary atherosclerosis. Lai et al. [95,96,97] also showed this association in their earlier studies. Similarly, Lucas et al. [92] found greater carotid plaque formation at baseline among cocaine users compared with cocaine nonusers. This variability in findings across studies regarding association between cocaine use and subclinical CAD might be explained by different CAD risk factor profiles of the study populations. The studies reporting cocaine-associated plaques included predominantly African American participants, of whom 40% to 100% were HIV-positive [91,92,100,101,102]. In contrast, all other studies showing no association between cocaine use and coronary calcifications did not include any HIV-positive individuals [84,88,89,90]. It has been widely known that HIV infection is a risk factor for CAD. Therefore, the study subjects with HIV may have been at higher risk for development of CAD, as was pointed by Arora et al. [88].

### 3.3. Effects of Cocaine on Mortality

Several studies estimated cardiovascular mortality among cocaine users. These studies have shown mixed results with regard to association of cocaine use with cardiovascular mortality. Some studies have reported higher risk for cardiovascular mortality among cocaine users compared with cocaine non-users. For example, DeFilippis et al. [93] retrospectively analyzed records of patients with MI at ≤50 years of age between 2000 and 2016 to examine the risk of cocaine use for cardiovascular mortality and all-cause mortality. Patient data were obtained from two large academic medical centers in the US There were 99 individuals in the cocaine-group (mean age: 44 (40–46), 85% male) and 1873 individuals in the non-cocaine group (mean age: 45 (42–48), 80% male). The authors found significant associations of cocaine use with cardiovascular mortality (HR=2.32, 95% CI: 1.11-4.85) and all-cause mortality (HR = 1.91, 95% CI: 1.11–3.29). In Spain, Morentin et al. [94] investigated the prevalence of recent cocaine use in individuals who had died by sudden cardiovascular death (SCVD) between January 2003 and December 2009 (*n* = 311). The mean age was 41 ± 7 years, and 82% were male. Individuals who had died by sudden deaths not due to cardiovascular diseases (SnoCVD) served as the control group (*n* = 126). The average age and percentage of males in the control group were 39 ± 7 years and 71%. The authors found that recent cocaine use was a significant risk factor for SCVD (OR = 4.10, 95% CI: 1.12–15.0). In contrast, Qureshi et al. [95] found that regular cocaine use was not associated with cardiovascular mortality (relative risk (RR) = 0.6, 95% CI: 0.1–4.7). The study subjects in this retrospective study were civilian non-institutionalized US adults aged 18–45 in the NHANES dataset. The study included 7751 cocaine nonusers (mean age: 31 ± 8 years, 43% males) and 178 regular cocaine users (lifetime cocaine use > 100 times) (mean age: 33 ± 7 years, 70% males). Although the study results showed a significant association between regular cocaine use and all-cause mortality (RR = 1.9, 95% CI: 1.2–3.0), regular cocaine use was not associated with cardiovascular mortality.

Prior studies examining the association of cocaine use with all-cause mortality have also reported inconsistent findings. In some studies, cocaine use was significantly associated with all-cause mortality. As mentioned previously, DeFilippis et al. [93] and Qureshi et al. [95] found an approximately two-fold higher all-cause mortality among cocaine users compared with cocaine nonusers. Similarly, Hser et al. [96] found an elevated mortality risk associated with cocaine use relative to methamphetamine use (HR = 3.56, 95% CI: 1.95–6.48). The subjects in this study were women admitted to drug abuse treatment programs in the US between 2000 and 2002. Contrary to the findings of these studies, some studies have reported no significant association between in-hospital mortality and cocaine use [84,97]. Atoui et al. [97] conducted a retrospective chart review of patients admitted with chest pain to a US-based hospital between July 2009 and June 2010. Of the study population with no risk factors for CAD, 54 were cocaine users (mean age = 44 ± 10 years, 59% males) and 372 were cocaine non-users (mean age = 43 ± 12 years, 49% males). The study results showed no significant differences in length of stay and in-hospital mortality between cocaine users and nonusers. Similarly, in the aforementioned study by Gupta et al. [84] in-hospital mortality was not significantly different between the cocaine group and the non-cocaine group (OR = 1.00, 95% CI: 0.69–1.44). As mentioned previously, in the study by Gunja et al. [87] cocaine use was initially found to be significantly associated with 1-year all-cause mortality after adjusting for cardiac risk factors and risky behaviors among veterans with CAD (HR = 1.22, 95% CI: 1.04–1.42) [87]. However, after controlling for causal pathway conditions, mortality was no longer significantly associated with cocaine use (HR: 1.15, 95% CI: 0.99–1.33).

In summary, some prior studies have reported an association between cocaine use and cardiovascular or all-cause mortality [93,94,96]. However, this association was not observed in other studies [84,97]. Variations in findings across studies may be driven by heterogeneity in patient characteristics (e.g., age), risky behaviors (e.g., smoking, alcohol, or other illicit drug use), and traditional risk factors (e.g., morbidities), all of which are predictors of mortality. Indeed, the study by Gunja et al. [87] showed how the association between cocaine use and mortality was confounded by these factors. In their study, cocaine was initially found to be associated with increased all-cause mortality. However, this association was no longer observed after controlling for causal pathway conditions such as congestive heart failure, cardiogenic shock, dialysis, depression, anxiety, ACS, and clinical status. This finding suggests that the effects of cocaine on mortality are largely dependent on individual clinical risk factors. Notably, mortality risk was not significantly higher among cocaine users if the cocaine users had fewer risk factors compared with cocaine nonusers. For example, subjects in the studies performed by Atoui et al. [97] and Gupta et al. [84] were individuals at low risk for CVD and young adults with few CV risk factors, respectively. Both studies found no association between cocaine use and mortality. In contrast, the subjects in studies reporting an association between cocaine use and mortality were at higher risk. The presence of risk factors is likely to augment the risk of mortality following cocaine use. Furthermore, frequency of cocaine use could be an important factor affecting mortality risk among cocaine users. As observed in the study by Qureshi et al., all-cause mortality was about two times higher among regular cocaine users (lifetime cocaine use > 100 times) compared with cocaine nonusers [95]. However, all cause-mortality of infrequent cocaine users (lifetime cocaine use: 1–10 times) or frequent cocaine users (lifetime cocaine use > 10 times) was not significantly different from that of cocaine nonusers in this study.

## 4. Cocaine and Nutrition

Cocaine use/abuse often affects food intake behavior and suppresses appetite, which may lead to the disruption of metabolic and neuroendocrine regulation. In addition, cocaine-induced malnutrition may decrease levels of neurotransmitters, and alter amino acid absorption and utilization. As such, chronic exposure to cocaine can result in an increased risk of health conditions such as hypertension, body weight problems, diabetes, and metabolic syndrome.

Cocaine affects appetite and body weight through multifactorial mechanisms. As mentioned previously, cocaine inhibits the reuptake of dopamine by interacting with the dopamine transporter, resulting in increased levels of dopamine in the central nervous system. Subsequently, changes in dopamine levels affect eating behavior and body weight [103,104,105]. Increased dopaminergic neurotransmission suppresses overall food intake whereas it increases fat-rich food intake [106]. In addition, cocaine blocks the reuptake of serotonin by interacting with the serotonin transporter, inducing leptin-dependent anorexic effect [107,108]. Prior studies demonstrated that cocaine also upregulated neuromodulators such as cocaine- and amphetamine-regulated transcript (CART), which plays an important role in regulating food intake, maintaining body weight, and in endocrine and cardiovascular functions [109,110]. Overexpression of CART has been reported to decrease food intake and change lipid metabolism related to fat storage [111,112].

In accordance with these mechanisms, several pre-clinical studies have shown the effects of cocaine on food consumption and the nutritional status in animals [113,114,115]. For example, Balopole et al. [113] reported a decrease in food intake after cocaine administration to rats (10, 15, and 25 mg/kg). They found that the cocaine-induced anorexia was transient and dose-dependent. After an hour of anorexic effect, it was shown that animals overconsumed foods. Therefore, total food intake was not significantly different between cocaine- and saline-exposed rats. Another study examined the effects of cocaine on the milk intake and body weight in rats [114]. Findings of this study suggested that cocaine disrupted ingestion primarily by interfering with the appetitive phase of feeding behavior (orientation and approach to food) rather than the consummatory phase (ingestion of food). A study by Church et al. [115] examined the effects of prenatal cocaine exposure on maternal/fetal toxicity in animals. Cocaine treatments in rats (20, 30, 40, and 50 mg/kg) resulted in significant reductions in the maternal weight gain and food consumption in a dose-dependent manner. Undernutrition led to a significant reduction in fetal weight. However, maternal water consumption was significantly increased in the cocaine-exposed animals possibly because of the increased locomotor activity and diuretic effect. Furthermore, cocaine provoked diarrhea in some of animals that received high doses, suggesting that cocaine, as a gastrointestinal irritant, might cause malabsorption and loss of electrolytes and nutrients, which ultimately can lead to malnutrition.

Human studies have also shown cocaine’s anorexigenic effects and the resulting weight reduction in cocaine users [116,117]. Low caloric intake, together with abnormal metabolic and gastrointestinal functions, can lead to malnutrition among cocaine users [118]. For example, Escobar et al. [119] found that hemoglobin and hematocrit levels in cocaine users were below normal, indicating protein-energy malnutrition and anemia. As the authors pointed out, anemia in this population might be associated with a diet poor in micronutrients (e.g., iron), inadequate protein consumption, and clinical issues such as decreased intrinsic factor secretion, intestinal perforations, and bacterial or infectious diseases. Indeed, three cases were reported where patients required surgery for their intestinal perforations after cocaine use [120]. Cocaine led to mesenteric vasoconstriction and focal tissue ischemia by blocking the reuptake of norepinephrine, which might lead to intestinal perforations. Cocaine users in the study by Escobar et al. were also found to have altered lipid and glucose profiles, with low levels of high density lipoprotein (HDL) cholesterol and high levels of triglycerides, LDL cholesterol, total cholesterol, and glucose. These findings suggested that cocaine users might be at a high risk for metabolic and cardiovascular problems. Of note, cocaine users did not experience weight gain despite a compensatory increase in fat consumption following the cocaine-induced anorexia [117]. However, the cessation of cocaine use resulted in weight gain [117,121]. In a study by Ersche et al., cocaine users consumed significantly more fatty foods and carbohydrates compared with cocaine nonusers, but there was no concomitant weight increase in the cocaine group [117]. The authors suggested that an imbalance between fat intake and storage could lead to weight gain among cocaine users when they stop using cocaine. This imbalance might result from metabolic alterations from repeated cocaine use. It is well-known that weight gain increases the risk of cardio-metabolic disorders such as diabetes and cardiovascular conditions [122]. Therefore, weight control, as a means to prevent and lessen cardiovascular diseases, has profound implications during cocaine abstinence.

In summary, cocaine use affects eating behavior and suppresses appetite, leading to malnutrition and anorexia through disruption of the metabolic process and neuroendocrine regulation. Also, cocaine uptake in the body can lead to mesenteric vasoconstriction and focal tissue ischemia, and alter lipid as well as glucose profiles, presumably resulting in increased risk for metabolic and cardiovascular problems in cocaine users. Notably, the cessation of cocaine use causes sudden/excess weight gain during the recovery period/process, leading to increased cardiovascular and cardio-metabolic risks. As such, cocaine-induced changes in food intake patterns and the metabolic process can lead to cardiovascular complications during addiction as well as cessation periods.

## 5. Conclusions

Cocaine use/abuse has been known to make changes in nutrient status and metabolism, which can result in an increased risk of long-term health conditions including eating disorders, metabolic syndrome, and psychological abnormalities. In this review, we focus the deleterious acute and chronic effects of cocaine use particularly on cardiovascular outcomes. We summarized the pathophysiological mechanisms of cocaine on cardiovascular health, which were multifactorial and complex. Compared to chronic effects, acute effects of cocaine have been well-characterized in previous studies. Use of cocaine, a potent cardiovascular stimulant, has been associated with electrocardiographic abnormalities, elevated blood pressure, arrhythmia, and acute MI. The risk of MI among cocaine users was particularly influenced by individuals’ cardiac risk factors and risky behaviors. Cocaine use can lead to acute conditions in a multifactorial fashion, for example, by blocking sodium/potassium channels in the heart and enhancing coronary artery spasm/vasoconstriction in vessels. In contrast, chronic effects of cocaine are difficult to determine as evidenced by inconsistent findings across previous studies. Some studies have reported an association of chronic cocaine use with coronary atherosclerosis using coronary calcification as a marker. Conversely, other studies have demonstrated no association between chronic cocaine use and coronary calcification. Of note, the subjects included in studies showing this association were at higher risk for CAD compared with those in the studies that reported no association. Therefore, chronic effects of cocaine may have been more prominent among those with higher CAD risk factor profiles. Contributions of cocaine to chronic conditions were also multifaceted. Long-term exposure to cocaine can exert chronic effects, for example, on the heart through non-ischemic myocardial depression and vessels by inducing endothelial cell injury and intracoronary thrombosis. Furthermore, prior studies suggested that risky behaviors, risk factors for CVD, and frequency of cocaine use may contribute to association between cocaine use and mortality. To evaluate the effects of long-term cocaine use on atherosclerosis and mortality more precisely, large, well-designed longitudinal studies are required with subjects from both low and high-risk populations. β-blocker therapy has often been suggested for cocaine users, in particular, for those with cocaine-associated heart failure. Studies have shown β-blockers lowered blood pressure, improved LV ejection fraction, and reduced the incidence of MI and mortality among cocaine users [123,124,125]. Understanding the multifactorial pathophysiological mechanisms of cocaine could help clinicians recognize the various symptoms after cocaine use/abuse and improve treatment of patients with either acute or chronic symptoms. The various deleterious CV outcomes resulting from cocaine use highlight the need for education regarding adverse cardiac effects of cocaine use, and access to effective interventions for cocaine abusers. Concurrently, alterations in lifestyle and behaviors (e.g., alcohol abuse or tobacco use) are also important for reducing the harmful adverse cardiac effects that these behavioral factors contribute to among cocaine users.

## Figures and Tables

**Table 1:** Acute and chronic effects of cocaine on the cardiovascular system.

Study (year)	Country	Study Design	Data Source	Study Population (Sample Size)	Male %, Age (mean ± SD)	Outcome(s)	Findings
**Acute effects of cocaine**							
Kozor et al. (2014) [81]	Australia	Cross-sectional	Study participants	Adults with no coronary disease, no previous MI, no contraindication to CMR imaging, and no cocaine use in the 48 h prior to image acquisition (*n* = 20 for social cocaine users; *n* = 20 for cocaine non-users)	85%, 37 ± 7 yrs in the social cocaine users’ group; 95%, 33 ± 7 yrs in the cocaine nonusers group	Systolic blood pressure, aortic stiffness, and LV mass	Cocaine use associated with high systolic blood pressure (134 ± 11 vs. 126 ± 11 mmHg), increased aortic stiffness, and greater LV mass (124 ± 25 vs. 105 ± 16 g) compared with no cocaine use
Sharma et al. (2016) [43]	US	Retrospective	ECG recordings in the Atherosclerosis Risk in Communities (ARIC) study from Aug. 2006 to Dec. 2014	Cocaine-dependent subjects (*n* = 97); non-cocaine-using control subjects (*n* = 8513)	86%, 50 ± 4 yrs in the cocaine-dependent subjects’ group; 46%, 52 ± 5 yrs in the controls group	Resting ECG parameters	Significant effects of cocaine use on early repolarization (OR = 4.92, 95% CI: 2.73–8.87), bradycardia (OR = 3.02, 95% CI: 1.95-4.66), severe bradycardia (OR = 5.11, 95% CI: 2.95-8.84), and heart rate (B weight = −5.84, 95% CI: −7.85 to −3.82)
Kariyanna et al. (2018) [82]	US	Case-report	Patient	A 55-year-old woman presenting with a chest pain after cocaine use (*n* = 1)	0%, 55 yrs	Second degree Mobitz type II atrioventricular block	Cocaine-induced Mobitz type II second degree atrioventricular block
Satran et al. (2005) [83]	US	Retrospective	Angiographic database at Hennepin County Medical Center in Minnesota	Patients with a history of cocaine use (*n* = 112); Patients with no history of cocaine use (*n* = 79)	79%, 44 ± 8 yrs in the cocaine users’ group; 61%, 46 ± 5 yrs in the cocaine non-users group	CAA	Significantly higher CAA in cocaine users compared with cocaine nonusers (30.4% vs. 7.6%)
Gupta et al. (2014) ^1^ [84]	US	Retrospective	Acute Coronary Treatment and Intervention Outcomes Network Registry-Get With The Guidelines (ACTION Registry-GWTG)	Patients admitted within 24 h of acute MI from July 2008 to March 2010 (*n* = 924 in the cocaine group; *n* = 102,028 in the non-cocaine group)	80%, 50 (range: 44–56) yrs in the cocaine group; 65%, 64 (range: 54–76) yrs in the non-cocaine group	Acute STEMI, cardiogenic shock, multivessel CAD, and in-hospital mortality	Higher percentages of STEMI (46.3% vs. 39.7%) and cardiogenic shock (13% vs. 4.4%) in the cocaine group, but a lower percentage of multivessel coronary artery disease (53.3% vs. 64.5%). Similar in-hospital mortality between the cocaine group and the non-cocaine group (OR = 1.00, 95% CI: 0.69–1.44)
Salihu et al. (2018) [85]	US	Retrospective	National Inpatient Sample (NIS) from Jan. 2002 to Dec. 2014	Pregnant women aged 13-49 yrs who had pregnancy-related inpatient hospitalizations (*n* = 153,608 cocaine users; *n* = 56,882,258 non-drug users)	0%, Age group: 13–24 (21.4%); 25–34 (55.4%); 35–49 (20.5%) in the cocaine users’ group; 0%, Age group: 13-24 (34.0%); 25–34 (51.3%); 35–49 (14.7%) in the non-drug users’ group	Acute MI or cardiac arrest	Cocaine use associated with acute MI or cardiac arrest (adjusted OR = 1.83, 95% CI: 1.28–2.62)
Aslibekyan et al. (2008) [86]	US	Retrospective	National Health and Nutrition Examination Survey (NHANES) in 1988–1994 and 2005–2006	Civilian non-institutionalized US adults (a) aged 18-59 (*n* = 11,993); (b) aged 18-45 (*n* = 9337)	(a) 46%, 36 yrs (N/R); (b) 39%, 31 yrs (N/R)	Prevalence of MI	(a) No significant association between cocaine use and MI in the 18–59 age group; (b) Significant association between cocaine use of > 10 lifetime instances and MI in the 18–45 age group (aged-adjusted OR = 4.60, 95% CI: 1.12–18.88), but this association was attenuated in the multivariate-adjusted model (OR = 3.84, 95% CI: 0.98–15.07)
Gunja et al. (2018) ^2^ [87]	US	Retrospective	Veterans Affairs database	Veterans with CAD undergoing cardiac catheterization from Oct. 2007 to Sep. 2014 (*n* = 3082 in the cocaine group; *n* = 118,953 in the non-cocaine group)	98.6%, median age: 58 (IQR: 54–62) yrs in the cocaine group; 98.6%, median age: 65 (IQR: 61–72) yrs in the non-cocaine group	MI and 1-year all-cause mortality	With adjustment of basic cardiac risk factors, cocaine use was significantly associated with MI (HR = 1.40, 95% CI: 1.07–1.83) and mortality (HR = 1.23, 95% CI: 1.08–1.39). After adjustment for risky behaviors, cocaine use was associated with mortality (HR = 1.22, 95% CI: 1.04–1.42), but not with MI (HR = 1.17, 95% CI: 0.87–1.56). After adjustment for causal pathway conditions, mortality was no longer significant (HR = 1.15, 95% CI: 0.99–1.33)
**Chronic effects of cocaine**							
Maceira et al. (2014) [45]	Spain	Prospective	Study participants and a gender and age matched healthy group	Cocaine abusers attending a rehabilitation clinic for the first time (*n* = 94)	86%, 37 ± 7 yrs	Cocaine cardiotoxicity using a CMR protocol	Increased LV end-systolic volume, LV mass index, and RV end-systolic volume, and decreased LV ejection fraction and RV ejection fraction in cocaine abusers compared with those in the gender and age matched healthy group
Arora et al. (2015) [88]	US	Cross-sectional	Drug treatment center in Florida	Caucasian adults with cocaine use disorder (*n* = 33)	33%, 37 ± 9 yrs	Presence of subclinical CAD using CIMT	No association between chronic cocaine use and subclinical CAD measured by CIMT
Bamberg et al. (2009) [89]	US	Nested matched cohort	Massachusetts General Hospital	Patients who presented to the emergency department with acute chest pain in May to July, 2005 (*n* = 44 in the cocaine group; *n* = 132 in the non-cocaine group)	86%, 46 ± 7 yrs in the cocaine group; 86%, 46 ± 7 yrs in the non-cocaine group	ACS and CAD using coronary CT	Significant association of cocaine use with increased risk of ACS group (OR = 5.79, 95% CI: 1.24–27.02), but no association with coronary stenosis
Chang et al. (2011) [90]	US	Cross-sectional	University of Pennsylvania Hospital	Patients who received coronary CTA for evaluation of CAD in the emergency department from May 2005 to Dec. 2008 (*n* = 157 in the cocaine group; *n* = 755 in the non-cocaine group)	58%, 46 ± 6 yrs in the cocaine group; 40%, 48 ± 9 yrs in the non-cocaine group	CAD	No association between recent cocaine use and the presence of coronary lesions ≥ 25% (adjusted RR = 0.92, 95% CI: 0.58–1.45) and coronary lesions ≥ 50% (adjusted RR = 0.96, 95% CI: 0.46–2.01)
Lai et al. (2016) [91]	US	Cross-sectional	Study participants	African American adults with/without HIV infection in Baltimore (*n* = 737 in the cocaine group; *n* = 692 in the non-cocaine group)	60.3%, 45 (IQR: 40–50) yrs in the entire population	Subclinical CAD defined by the presence of CAC detected by noncontrast CT and/or coronary plaque detected by contrast-enhanced coronary CT angiography	Chronic cocaine use associated with high risk for subclinical CAD (propensity score-adjusted prevalence ratio = 1.27, 95% CI: 1.08–1.49), CAC (propensity score-adjusted prevalence ratio=1.26, 95% CI: 1.05–1.52), any coronary stenosis (propensity score-adjusted prevalence ratio = 1.30, 95% CI: 1.08–1.57), and calcified plaques (propensity score-adjusted prevalence ratio = 1.37, 95% CI: 1.10–1.71)
Lucas et al. (2016) [92]	US	Cross-sectional and longitudinal	Study participants	Adults with/without human immunodeficiency virus infection in Baltimore (*n* = 57 never cocaine users; *n* = 82 past cocaine users; *n* = 153 current cocaine users)	67%, 46 (IQR: 41–53) yrs in the never users; 66%, 51 (IQR: 46–54) yrs in the past users; 75%, 49 (IQR:45–52) yrs in the current users	Subclinical CVD: carotid artery plaque	Cocaine use associated with approximately three-fold higher odds of carotid plaques at baseline (OR = 3.3, 95% CI: 1.5–7.3 for past cocaine users vs. cocaine nonusers; OR = 2.7, 95% CI: 1.3–5.5 for the current cocaine users vs. cocaine nonusers)
**Cocaine effects on mortality**							
DeFilippis et al. (2018) [93]	US	Retrospective cohort	Two academic medical centers (Brigham and Women’s Hospital and Massachusetts General Hospital)	Patients presenting with an MI at ≤50 years between 2000 and 2016 (*n* = 99 in the cocaine group; 1873 in the non-cocaine group)	85%, 44 (range: 40–46) yrs in the cocaine group; 80%, 45 (range: 42–48) yrs in the non-cocaine group	Cardiovascular mortality and all-cause mortality	Significant association of cocaine use with cardiovascular mortality (HR = 2.32, 95% CI: 1.11–4.85) and all-cause mortality (HR = 1.91, 95% CI: 1.11–3.29)
Morentin et al. (2014) [94]	Spain	Case-control retrospective	Forensic autopsy reports in Biscay, Spain	All SCVD in individuals aged 15–49 (*n* = 311); SnoCVD (*n* = 126) from Jan. 2003 to Dec. 2009	82%, 41 ± 7 yrs in SCVD; 71%, 39 ± 7 yrs in SnoCVD	Cocaine detected in blood	Cocaine being the risk for SCVD (OR = 4.10; 95% CI: 1.12–15.0)
Qureshi et al. (2014) [95]	US	Retrospective	NHANES in 1988-1994	Civilian non-institutionalized US adults aged 18–45 (*n* = 7751 cocaine nonusers; *n* = 730 infrequent cocaine users (1–10 times); *n* = 354 frequent cocaine users (>10 times); *n* = 178 regular cocaine users (>100 times))	43%, 31 ± 8 yrs in the cocaine non-users’ group; 59%, 31±10 yrs in the infrequent cocaine users group; 65%, 33 ± 9 yrs in the frequent cocaine users group; 70%, 33 ± 7 yrs in the regular cocaine users group	Cardiovascular mortality and all-cause mortality	Regular lifetime cocaine use was associated with high all-cause mortality (RR = 1.9, 95% CI: 1.2–3.0), but not cardiovascular mortality (RR = 0.6, 95% CI: 0.1–4.7) compared with cocaine nonusers
Hser et al. (2012) [96]	US	Prospective cohort	California Treatment Outcome Project (CalTOP) between 2000 and 2002, the National Death Index by 2008, the National Death Register by 2010, and the California Department of Mental Health	Women admitted to 40 drug abuse treatment programs through CalTOP (*n* = 4,253 for those alive in 2010; *n* = 194 for those deceased by 2010)	0%, 33 ± 8 yrs for living; 0% 37 ± 7 yrs for the deceased	8 to 10-year mortality	Cocaine was associated with higher mortality relative to methamphetamine (HR = 3.56, 95% CI: 1.95–6.48)
Atoui et al. (2011) [97]	US	Retrospective chart review	Electronic medical records in Bronx Lebanon Hospital Center	Patients admitted with chest pain to the hospital who had no cardiovascular risk factors from July 2009 to June 2010 (*n* = 54 in the cocaine group; *n* = 372 in the non-cocaine group)	59%, 44 ± 10 yrs in the cocaine group; 49%, 43 ± 12 yrs in the non-cocaine group	Length of stay and mortality	No significant difference in length of stay (3.0 vs. 2.4) and in-hospital mortality (0% vs. 1%) between the cocaine group and the non-cocaine group

ACS: Acute coronary syndrome; CAA: Coronary artery aneurysm; CAC: Coronary artery calcium; CAD: Coronary artery disease; CI: Confidence interval; CIMT: Carotid intima media thickness; CMR: Cardiovascular magnetic resonance; CT: Computed tomography; CTA: Computerized tomographic angiography; CVD: Cardiovascular disease; ECG: Electrocardiogram; HR: Hazard ratio; IQR: Interquartile range; LV: Left ventricular; MI: Myocardial infarction; N/R: not reported; OR: Odds ratio; RR: Relative risk; RV: Right ventricular; SCVD: Sudden cardiovascular death; SnoCVD: Sudden death not due to cardiovascular diseases; STEMI: ST elevation myocardial infarction. ^1^ Including acute effects (i.e., acute STEMI and cardiogenic shock) and chronic effect (i.e., multivessel CAD) of cocaine and mortality as the study outcomes. ^2^ Including acute effect of cocaine (i.e., MI) and 1-year all-cause mortality as the study outcomes.
